# Comment on Burgos et al. Fusionless All-Pedicle Screws for Posterior Deformity Correction in AIS Immature Patients Permit the Restoration of Normal Vertebral Morphology and Removal of the Instrumentation Once Bone Maturity Is Reached. *J. Clin. Med.* 2023, *12*, 2408

**DOI:** 10.3390/jcm12144677

**Published:** 2023-07-14

**Authors:** Justin V. C. Lemans, Tom P. C. Schlösser, René M. Castelein, Moyo C. Kruyt

**Affiliations:** Department of Orthopedic Surgery, University Medical Center Utrecht, P.O. Box 85500, 3508 GA Utrecht, The Netherlands; j.v.c.lemans-3@umcutrecht.nl (J.V.C.L.); t.p.c.schlosser@umcutrecht.nl (T.P.C.S.); r.m.castelein@umcutrecht.nl (R.M.C.)

With great interest, we read the recently published paper “Fusionless All-Pedicle Screws for Posterior Deformity Correction in AIS Immature Patients Permit the Restoration of Normal Vertebral Morphology and Removal of the Instrumentation Once Bone Maturity is Reached” by Burgos et al. [1]. The authors demonstrated that adolescent idiopathic scoliosis (AIS) patients with flexible curves can be treated with temporary posterior instrumentation including the lumbar spine. The authors presented 36 skeletally immature AIS patients with an average major Cobb angle of 53.7°, which corrected to 5.5°. After a mean of 31 months, the instrumentation was removed. Two years after removal, the Cobb angle was 13.1° and the lumbar spine was mobile with a range of motion of 51°.

We applaud the authors for investigating this novel strategy. Obviously, and as also mentioned by the authors, this strategy can only be used in selected patients with flexible curves that are moderate in size and with a maturity status of Risser 0–3 to harness remaining growth modulation. Despite the promising results, we highlight the limitations of the methods used for the assessment of growth modulation, and think that a potential danger of this strategy is secondary progression of the curve.

The authors attribute the absence of curve progression to the bony remodeling around the apex. They report a decrease in coronal apical wedging over time. Two-dimensional measurements of vertebral wedging, however, are highly influenced by spinal rotation and tilt [2,3,4]. Consequently, true vertebral remodeling over time should be assessed in the exact same 2D plane. Instead of comparison of the final apical wedging to the wedging of the rotated apex preoperatively, a comparison to the immediate postoperative state would have been preferable to mitigate the effect of differences in orientation and assess the true vertebral growth.

With respect to risk of curve progression, previous reports, where implants were removed 2–4 years after fusion surgery, showed occasional progression of the main curve of around 5–25°, even in patients who had a solid fusion mass at time of implant removal [5,6,7]. In some patients, this also coincided with a major increase (>20°) in thoracic kyphosis [6]. Furthermore, in the growing population, Kocyigit et al. showed that removal of traditional growing rods at age 14 resulted in curve progression in 9/10 patients (pre-removal: 33°; post-removal: 40°; 2 years post-removal: 56°), which was a reason for definitive fusion in these patients [8].

We believe it is much more likely that the flexible curves and the aggressive correction may provide the conditions to allow for remodeling of the intervertebral disc (IVD), which plays a major role in the morphological changes observed in scoliosis [9]. The maturation of the discs in a(n) (almost) corrected position probably provides sufficient long-term stabilization, as is seen after bracing or in reduced non-structural curves. The question is whether the technique used in the current study (open surgery with full-density rigid immobilization of the spine) offers the right conditions for IVD function and health to remain. The authors report lumbar ROM >50°; however, they do not compare this to pre-operative levels, and they report no data on the size and shape of the IVD. Recent data from the EOS literature have shown, in both 2D and 3D, that prolonged fixation and/or intermittent lengthenings lead to height loss over time and induce degenerative changes in the IVDs [10,11]. Therefore, we strongly encourage the authors to follow up these patients in the long term.

In our opinion, regenerating the spine towards healthy alignment and function necessitates:

(1) Gradual correction of the spine in all three planes;
(2) Maintaining this correction for an extended period to allow for remodeling and maturation of both the vertebrae and discs;
(3) The above to be achieved without rigid immobilization of the spine to maintain healthy discs.

By using rigid instrumentation intended for stabilization to achieve spinal fusion, these goals become conflicting. More dynamic implants, which correct the curve while allowing motion, like an internal brace, may be more appropriate for this purpose; however, these have not yet been investigated in an AIS population [12,13].
